# Broadening the Photoluminescence Excitation Spectral Bandwidth of YVO_4_:Eu^3+^ Nanoparticles via a Novel Core-Shell and Hybridization Approach

**DOI:** 10.3390/ma12233830

**Published:** 2019-11-21

**Authors:** Jianhua Huang, Lu Tang, Nan Chen, Guoping Du

**Affiliations:** 1 School of Materials Science and Engineering, Nanchang University, Nanchang 330031, China; 2 Hunan Engineering Laboratory for Control and Optimization of PV Systems, Hunan Vocational Institute of Technology, Xiangtan 411104, China; 3 The Center of Collaboration and Innovation, Jiangxi University of Technology, Nanchang 330098, China

**Keywords:** lanthanide-doped YVO_4_, core-shell nanoparticle, inorganic–organic hybrid nanostructure, photoluminescence

## Abstract

For many optoelectronic applications, it is desirable for the lanthanide-doped phosphors to have broad excitation spectrum. The excitation mechanism of the lanthanide-doped YVO_4_, a high quantum efficient lasing material, primarily originates from the energy transfer process from the host VO_4_^3−^ complexes to the lanthanide ions, which has an excitation spectral bandwidth range of 230–330 nm. For applications in silicon solar cells, such phosphors can convert ultraviolet light to visible light for more efficient power generation, but this spectral range is still not broad enough to cover the entire ultraviolet spectrum of solar light. In this work, a novel core-shell and inorganic–organic hybridization strategy has been employed to fabricate Eu^3+^-doped YVO_4_ nanoparticles to broaden their photoluminescence excitation spectral bandwidth to the range of 230–415 nm, covering the entire ultraviolet spectrum of solar light and enabling their potential applications in silicon solar cells.

## 1. Introduction

Lanthanide ion-doped YVO_4_ (YVO_4_:Ln^3+^), such as YVO_4_:Nd^3+^, has been widely used for fabricating lasers [1,2]. Recently, luminescent YVO_4_:Ln^3+^ nanomaterials, such as YVO_4_:Eu^3+^ [3,4], YVO_4_:Eu^3+^,Bi^3+^ [5,6], YVO_4_:Yb^3+^ [7], YVO_4_:Yb^3+^,Bi^3+^ [8] etc., have received extensive attention for enhancing the conversion efficiency of solar cells. Solar cells, especially the silicon solar cells, usually have rather low quantum efficiency in the ultraviolet (UV) spectrum, but, in the visible/near infrared spectrum, however, their quantum efficiency is quite high. YVO_4_:Ln^3+^ nanomaterials are excited by UV lights of solar irradiance, and down-convert them to visible or near infrared lights. Through such down-conversion, the conversion efficiency of solar cells is consequently enhanced as a result of more efficient utilization of UV photons of solar irradiance [9,10]. 

The strong photoluminescence of YVO_4_:Ln^3+^ relies on the efficient energy transfer process from the host VO_4_^3-^ complexes to the Ln^3+^ [11], in which the VO_4_^3-^ is well excited by the UV lights. Such an energy transfer process effectively overcomes the drawback of low absorption cross sections of parity forbidden 4f–4f transitions of Ln^3+^ ions. The excitation spectrum of VO_4_^3−^ peaks at about 325 nm, and has a spectral range from 260 nm to 330 nm at the half maximum and a band edge at 350 nm [12]. This, however, is too narrow to cover the UV spectrum (250–400 nm) of the solar lights on earth surface for applications in solar cells. To extend its excitation spectrum, Bi^3+^ ions have been co-doped into YVO_4_:Ln^3+^ [5,6,8,13,14]. It was found that the peaking wavelength of its excitation spectrum is shifted to the longer wavelength at 342 nm and the band edge is extended to about 360 nm [8]. Up to now, however, the excitation bandwidth of YVO_4_:Ln^3+^ is still not wide enough to cover the UV spectrum of the solar irradiance. Previous work on LaF_3_:Eu^3+^ [15] has shown that its excitation spectral bandwidth can be effectively broadened by using a mixed photoluminescence sensitization from different types of organic ligands.

In this work, a novel strategy was employed to remarkably broaden the excitation spectrum of YVO_4_:Eu^3+^ nanoparticles through a dual–channel excitation approach, and the entire UV spectrum of the solar irradiance can be effectively covered. This was implemented by combining the two strong excitation channels, the efficient VO_4_^3−^→Eu^3+^ energy transfer after annealing and the photoluminescence sensitization by organic ligand after forming an inorganic–organic hybrid nanoparticle [16,17].

## 2. Experimental

**Figure 1:**
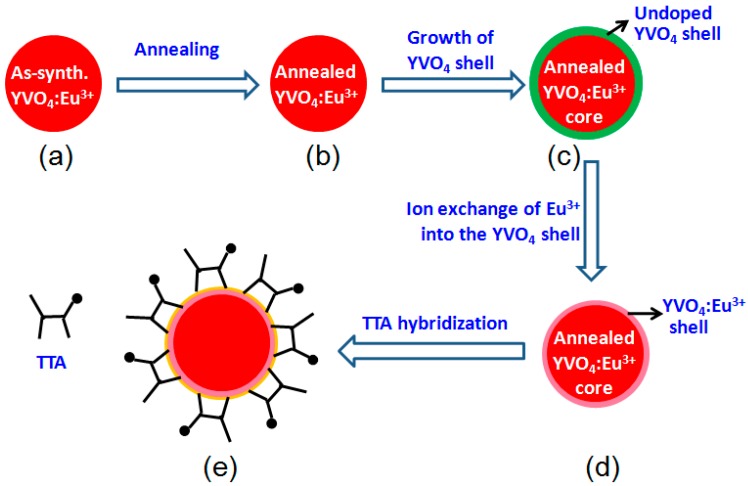
Illustration of preparation steps: (**a**) as-synthesized YVO_4_:Eu^3+^, (**b**) annealed YVO_4_:Eu^3+^, (**c**) core-shell YVO_4_:Eu^3+^@YVO_4_, (**d**) core-shell YVO_4_:Eu^3+^@YVO_4_:Eu^3+^, (**e**) hybrid YVO_4_:Eu^3+^@YVO_4_:Eu^3+^–TTA.

The experimental procedure is illustrated in Figure 1, and it is detailed in the following paragraphs.

### 2.1. Materials

Yttrium nitrate (Y(NO_3_)_3_·6H_2_O, 99.99 %), europium nitrate (Eu(NO_3_)_3_·6H_2_O, 99.99 %), sodium vanadate (Na_3_VO_4_·12H_2_O, AR), polyvinyl pyrrolidone (PVP K30, AR), ethylene glycol (EG, AR), anhydrous ethanol (AR), and 2-thenoyltrifluoroacetone (TTA, 98.0%) were used in this work. All were bought from Sinopharm Chemical Reagent Co., Ltd. (Shanghai, China). Deionized water was used throughout this work.

### 2.2. Synthesis and Annealing of YVO_4_:Eu^3+^ Nanoparticles

The YVO_4_:Eu^3+^ nanoparticles were synthesized using the solvothermal method [18]. First, 0.75 mmol Na_3_VO_4_·12H_2_O and 0.05 g PVP were dissolved in 8 mL ethylene glycol and 1 mL deionized water. Then, 9 mL of ethylene glycol solution containing 1 mmol Y(NO_3_)_3_·6H_2_O and 0.05 mmol Eu(NO_3_)_3_·6H_2_O were added to the above solution with vigorous magnetic stirring for 5 min. Then, 17 mL of de-ionized water was added to the above mixture solution, with stirring for another 5 min. The mixture solution was poured into a 50 mL Teflon-lined stainless steel autoclave and heated at 180 °C for 6 h to obtain YVO_4_:Eu^3+^ nanoparticles, and then naturally cooled to room temperature. The YVO_4_:Eu^3+^ nanoparticles were collected by centrifugation, washed with anhydrous ethanol for 3 times, and dried at 80 °C in air.

As shown later in this paper, the as-synthesized YVO_4_:Eu^3+^ nanoparticles possess only weak luminescence, which can be greatly enhanced by annealing. In this work, an annealing process at 800 °C for 2 h in air was conducted for the as-synthesized YVO_4_:Eu^3+^ nanoparticles for strong photoluminescence (Figure 1). It was found that their particle size only increased to some extent after the annealing, but they remained to be in nanometer scale. However, if the annealing temperature is over 800 °C, the YVO_4_:Eu^3+^ nanoparticles quickly grow to a much larger size. Similar behavior has been reported by others [19,20].

### 2.3. Synthesis of YVO_4_:Eu^3+^@YVO_4_ Core-Shell Nanoparticles

The annealed YVO_4_:Eu^3+^ nanoparticles (Section 2.2) were used as a core to grow a pristine YVO_4_ shell for forming the YVO_4_:Eu^3+^@YVO_4_ core–shell nanoparticles (Figure 1). The pristine YVO_4_ shell, which is homo-nanoparticled to the core, is expected to passivate the surface non-radiative traps or defects of the annealed YVO_4_:Eu^3+^ nanoparticles [21,22,23]. First, 0.75 mmol Na_3_VO_4_·12H_2_O and 0.05 g PVP were dissolved in 8 mL ethylene glycol and 1 mL deionized water. Second, 1 mmol of the annealed YVO_4_:Eu^3+^ nanoparticles were re-dispersed into 9 mL ethylene glycol under continuous ultrasonication for 30 min. The above two solutions were mixed, and appropriate amount of Y(NO_3_)_3_·6H_2_O was added to the mixture solution with vigorous magnetic stirring for 5 min. Third, 17 mL of de-ionized water was added to the above mixture solution under magnetic stirring for another 5 min. The mixture solution was poured into a 50 mL Teflon-lined stainless steel autoclave and heated at 200 °C for 2 h, and then naturally cooled down to room temperature. The resultant YVO_4_:Eu^3+^@YVO_4_ core–shell nanoparticles were collected by centrifugation, washed with anhydrous ethanol for 3 times, and dried at 80 °C in air. The molar ratios of the core to the shell (core/shell ratio) were 1:0.2, 1:0.25, 1:0.5, and 1:1. In this work, it was found when the core/shell ratio was 1:0.25, the YVO_4_:Eu^3+^@YVO_4_ core–shell nanoparticles had the highest photoluminescence intensity. Thus, the core/shell ratio of 1:0.25 was chosen for the following experimental steps.

### 2.4. Synthesis of YVO_4_:Eu^3+^@ YVO_4_:Eu^3+^ Nanoparticles

As shown in Figure 1, an ion exchange method was used to transform the as-synthesized YVO_4_:Eu^3+^@YVO_4_ core–shell nanoparticles (Section 2.3) to YVO_4_:Eu^3+^@YVO_4_:Eu^3+^ core–shell nanoparticles by following the procedures described previously [18]. Appropriate amount of Eu(NO_3_)_3_·6H_2_O was dissolved in 15 mL of anhydrous ethanol. The YVO_4_:Eu^3+^@YVO_4_ nanoparticles were added to above solution with ultrasonic processing for 30 min and then vigorous magnetic stirring for 1 h, the mixture solution was let to stand for 24 h. The resultant product was collected by centrifugation, washed with anhydrous ethanol for 3 times, and finally dried in air at 80 °C.

### 2.5. Synthesis of YVO_4_:Eu^3+^@YVO_4_:Eu^3+^–TTA Nanoparticles

At last, the YVO_4_:Eu^3+^@YVO_4_:Eu^3+^–TTA inorganic–organic hybrid nanoparticles were synthesized by the previously described procedures [17,18]. It was found in this work that both the as-synthesized YVO_4_:Eu^3+^ and the annealed YVO_4_:Eu^3+^ cannot form YVO_4_:Eu^3+^–TTA inorganic–organic hybrid structures. Only the ion exchanged YVO_4_:Eu^3+^ (Section 2.4) can readily chelate with TTA to form YVO_4_:Eu^3+^–TTA inorganic–organic hybrid structures [18]. The YVO_4_:Eu^3+^@YVO_4_:Eu^3+^ nanoparticles (Section 2.4) were added to 10 mL anhydrous ethanol with ultrasonic processing for 30 min. An appropriate amount of TTA was dissolved in 10 mL of anhydrous ethanol with ultrasonic processing for 15 min and then slowly dripped into the YVO_4_:Eu^3+^@YVO_4_:Eu^3+^ ethanol solution. The mixture solution was stirred for 1 h and then was left at rest for 12 h. The resultant product was collected by centrifugation, washed with anhydrous ethanol for 3 times and finally dried in air at 80 °C to obtain the YVO_4_:Eu^3+^@YVO_4_:Eu^3+^–TTA inorganic–organic hybrid nanoparticles. The amounts of TTA used in this step were 0.05, 0.1, 0.15, and 0.2 mmol.

### 2.6. Characterization

X-ray diffraction (XRD, Bruker D8 Focus, Karlsruhe, Germany) and transmission electron microscopy (TEM, JEM 2010, Japan) were used to investigate the crystal structures and microstructures of the nanoparticles. Their luminescence excitation and emission spectra were recorded with a Hitachi F-4600 fluorescence spectrophotometer, and their chemical bonding information was characterized by Fourier transform infrared (FTIR) spectroscopy (Nicolet 380, Thermo Fisher, USA). Their UV–Vis absorption spectra were monitored with a TU-1950 UV–Vis spectrophotometer. All measurements were conducted in air at room temperature.

## 3. Results and Discussion

### 3.1. Microstructural Characteristics

**Figure 2:**
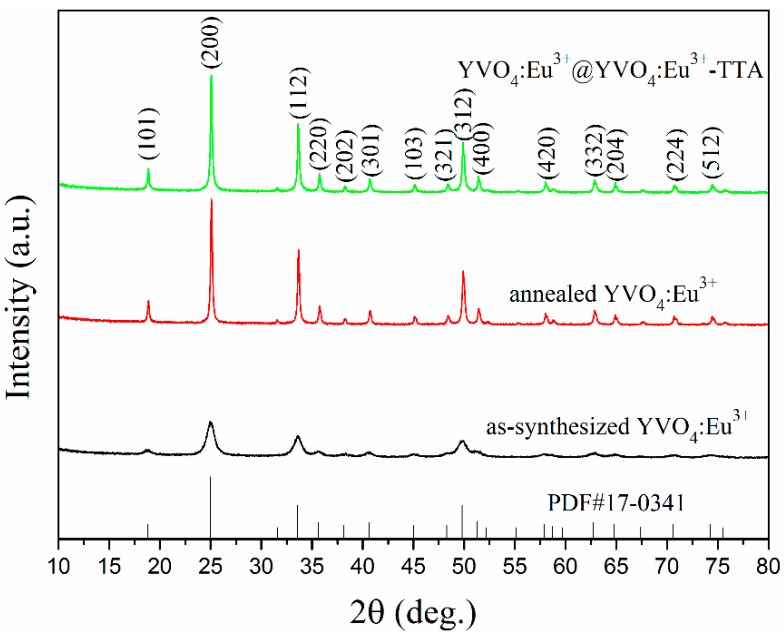
X-ray diffraction (XRD) patterns of the as-synthesized YVO_4_:Eu^3+^, the annealed YVO_4_:Eu^3+^ and the YVO_4_:Eu^3+^@YVO_4_:Eu^3+^–TTA inorganic–organic hybrid nanoparticles.

Figure 2 shows the X-ray diffraction (XRD, Bruker D8 Focus) patterns of the as-synthesized YVO_4_:Eu^3+^, the annealed YVO_4_:Eu^3+^ and the YVO_4_:Eu^3+^@YVO_4_:Eu^3+^–TTA hybrid nanoparticles. All of the diffraction peaks of samples are identical to the JCPDS 17-0341 of the tetragonal YVO_4_, and no impurity phases were present. The strong and narrow XRD peaks of the annealed YVO_4_:Eu^3+^ nanoparticles in Figure 2 suggested that the annealing process greatly enhanced their crystallization. It is also noted in Figure 2 that, after the core-shell process and hybridization with TTA, the YVO_4_:Eu^3+^ nanoparticles still had the same XRD patterns.

**Figure 3:**
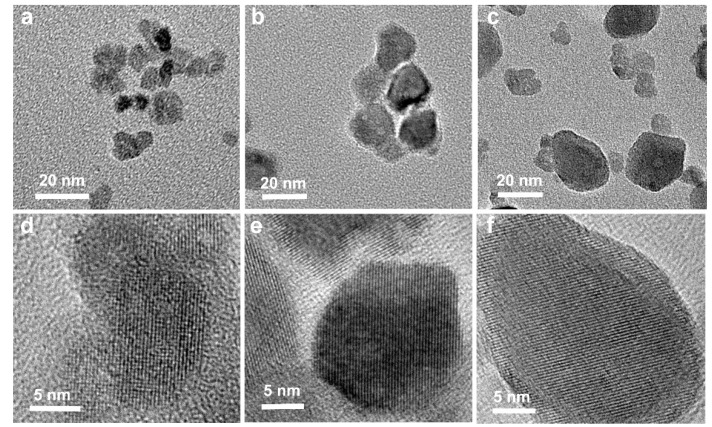
Transmission electron microscopy (TEM) (**a**–**c**) and HRTEM (**d**–**f**) images of the as-synthesized (unannealed) YVO_4_:Eu^3+^ nanoparticles (**a**,**d**), the annealed YVO_4_:Eu^3+^ nanoparticles (**b**,**e**) and the YVO_4_:Eu^3+^@YVO_4_:Eu^3+^ core-shell nanoparticles (**c**,**f**).

The microstructures of the as-synthesized and unannealed YVO_4_:Eu^3+^ nanoparticles, the annealed YVO_4_:Eu^3+^ nanoparticles and the YVO_4_:Eu^3+^@YVO_4_:Eu^3+^ core-shell nanoparticles are shown in Figure 3. The average particle sizes of the as-synthesized YVO_4_:Eu^3+^, the annealed YVO_4_:Eu^3+^, and the YVO_4_:Eu^3+^@YVO_4_:Eu^3+^ core-shell nanoparticles were about 15, 28, and 36 nm, respectively. As shown in Figure 3d–f, all of the nanoparticles were single crystals, and this may suggest that the YVO_4_ shell could be epitaxially grown on the annealed YVO_4_:Eu^3+^ core.

**Figure 4:**
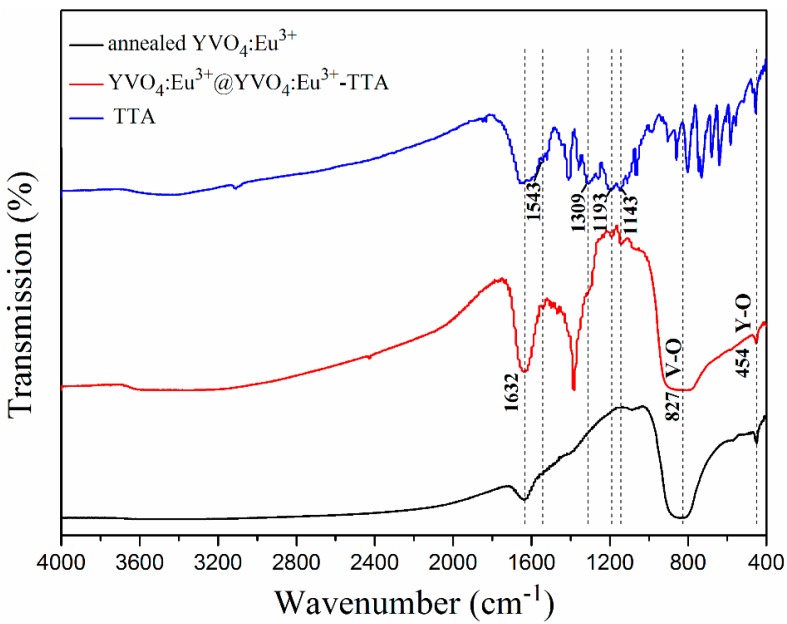
The Fourier transform infrared (FTIR) spectra of 2-thenoyltrifluoroacetone (TTA), the annealed YVO_4_:Eu^3+^ nanoparticles and YVO_4_:Eu^3+^@YVO_4_:Eu^3+^–TTA hybrid nanoparticles.

The Fourier transform infrared (FTIR, Nicolet 380) spectra of TTA, the annealed YVO_4_:Eu^3+^ and YVO_4_:Eu^3+^@YVO_4_:Eu^3+^–TTA hybrid nanoparticles are shown in Figure 4. The strong absorption band located at 1638 cm^−1^ is both from the H–O–H bending vibration of adsorbed water [24] and the ketonic group and thiophene of TTA [25]. Four new peaks at 1543 cm^−1^, 1309 cm^−1^, 1193 cm^−1^, and 1143 cm^−1^ for the YVO_4_:Eu^3+^@YVO_4_:Eu^3+^–TTA hybrid nanoparticles are attributed to the characteristic peaks of TTA [26]. This indicates that TTA successfully hybridized with the YVO_4_:Eu^3+^@YVO_4_:Eu^3+^ nanoparticles. The strong absorption peak at 827 cm^−1^ is due to the absorption of V–O bonds [27], while the weak peak at 454 cm^−1^ is due to the absorption of Y(Eu)-O bonds [28,29].

### 3.2. UV–Vis Absorption Spectra

**Figure 5:**
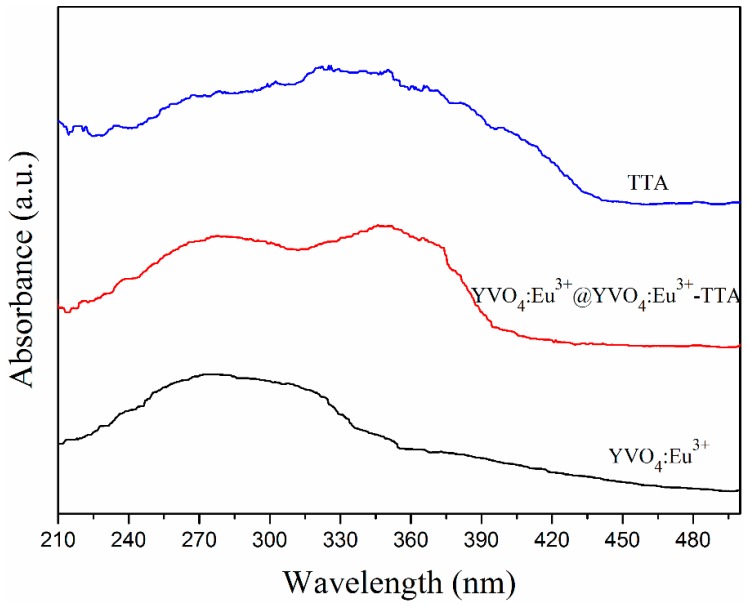
UV-Vis absorption spectra of the annealed YVO_4_:Eu^3+^ nanoparticles, TTA and YVO_4_:Eu^3+^@ YVO_4_:Eu^3+^–TTA hybrid nanoparticles.

The UV–Vis (TU-1950) absorption spectra of the annealed YVO_4_:Eu^3+^ nanoparticles, TTA, and YVO_4_:Eu^3+^@YVO_4_:Eu^3+^–TTA hybrid nanoparticles are shown in Figure 5. The broad absorption band (230–330 nm) of the annealed YVO_4_:Eu^3+^ nanoparticles was due to the VO_4_^3-^ groups in the host lattice of YVO_4_ nanoparticle [30]. It also can be seen in Figure 5 that TTA had a broad absorption band of 240–420 nm. Consequently, YVO_4_:Eu^3+^@YVO_4_:Eu^3+^–TTA nanoparticles possessed two broad absorption bands in the ultraviolet range, which is due to the presence of VO_4_^3^^−^ groups and TTA ligands.

### 3.3. Photoluminescence Properties

**Figure 6:**
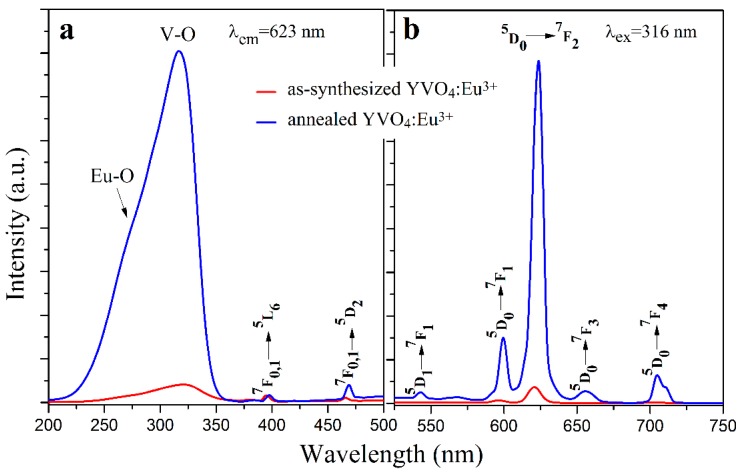
The excitation (**a**) and emission (**b**) spectra of the as-synthesized (unannealed) and the annealed YVO_4_:Eu^3+^ nanoparticles.

The photoluminescence excitation and emission spectra (Hitachi F-4600) of the as-synthesized (unannealed) YVO_4_:Eu^3+^ nanoparticles and the annealed YVO_4_:Eu^3+^ nanoparticles are shown in Figure 6. In Figure 6a, the strongest excitation band peaking at about 316 nm was a result of the charge transfer absorption of VO_4_^3-^ groups [31]. The minor hump-like peak at about 260 nm was due to the charge transfer band of Eu-O. The other weak excitation peaks at 397 nm (^7^F_0,1_→^5^L_6_) and 469 nm (^7^F_0,1_→^5^D_2_) were from Eu^3+^ ions [32]. In Figure 6b, all of the photoluminescence emissions were from the f–f transitions of Eu^3+^, including 542 nm (^5^D_1_→^7^F_1_), 598 nm (^5^D_0_→^7^F_1_), 623 nm (^5^D_0_→^7^F_2_), 655 nm (^5^D_0_→^7^F_3_), and 705 nm (^5^D_0_→^7^F_4_) [33].

After annealing, a remarkable enhancement in their photoluminescence was resulted. This is consistent with the previously reported results [19,21,34,35]. An increase of about 22 times in the emission of the YVO_4_:Eu^3+^ nanoparticles at 623 nm was observed after the annealing process. As shown in Section 3.1, the average particle size of the YVO_4_:Eu^3+^ nanoparticles increased from about 15 nm to 28 nm after annealing, but this size difference may not sufficiently explain such a remarkable increase of their photoluminescence. The XRD patterns in Figure 2 indicates that the crystallinity of the as-synthesized YVO_4_:Eu^3+^ nanoparticles was greatly improved by the annealing process. Furthermore, the number of crystal defects in the YVO_4_:Eu^3+^ nanoparticles as well as the non-radiative traps should also simultaneously decrease. These factors are mainly responsible for the notable enhancement of photoluminescence of the YVO_4_:Eu^3+^ nanoparticles by annealing.

**Figure 7:**
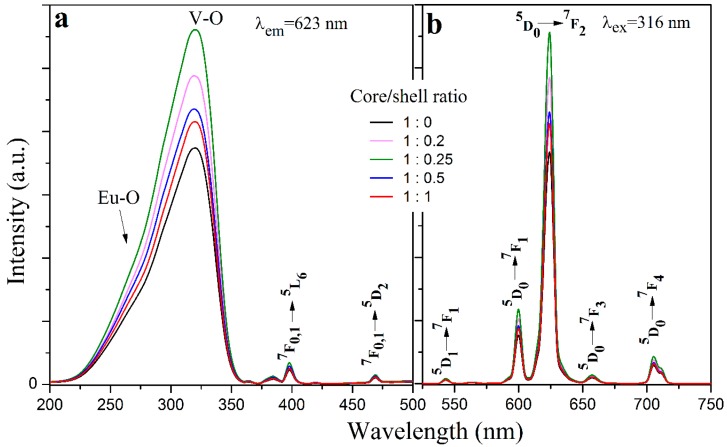
The excitation (**a**) and emission (**b**) spectra of the YVO_4_:Eu^3+^@YVO_4_ core-shell nanoparticles with different core/shell ratios.

Figure 7 shows the photoluminescence excitation and emission spectra of the YVO_4_:Eu^3+^@YVO_4_ core–shell nanoparticles having different core/shell ratios. Such a homo-nanoparticled shell is believed to be able to effectively passivate the non-radiative traps or defects on the surface of the core [19,20]. The photoluminescence spectra in Figure 7 are typical for the Eu^3+^-doped YVO_4_ phosphors. After the core-shell process, the YVO_4_:Eu^3+^@YVO_4_ nanoparticles had stronger photoluminescence than the unshelled YVO_4_:Eu^3+^ nanoparticles (core/shell ratio = 1:0 in Figure 7), indicating the passivation effect of the YVO_4_ shell. The YVO_4_:Eu^3+^@YVO_4_ core–shell nanoparticles with the core/shell ratio of 1:0.25 had the highest photoluminescence property (Figure 7).

**Figure 8:**
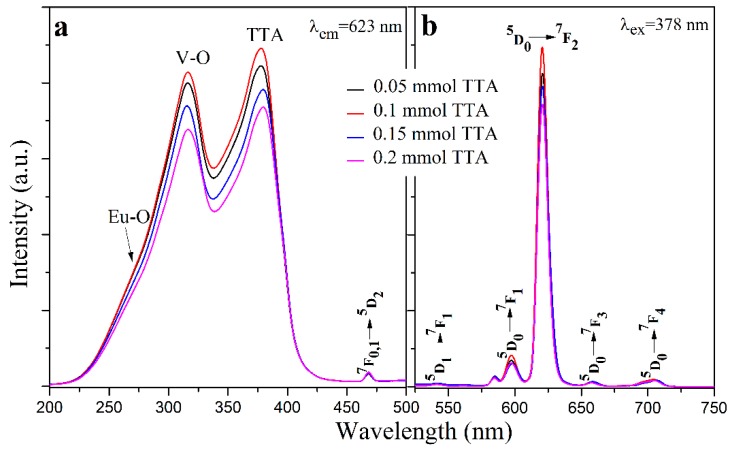
The photoluminescence excitation (**a**) and emission (**b**) spectra of YVO_4_:Eu^3+^@YVO_4_:Eu^3+^–TTA inorganic–organic hybrid nanoparticles prepared with different TTA amounts.

**Figure 9:**
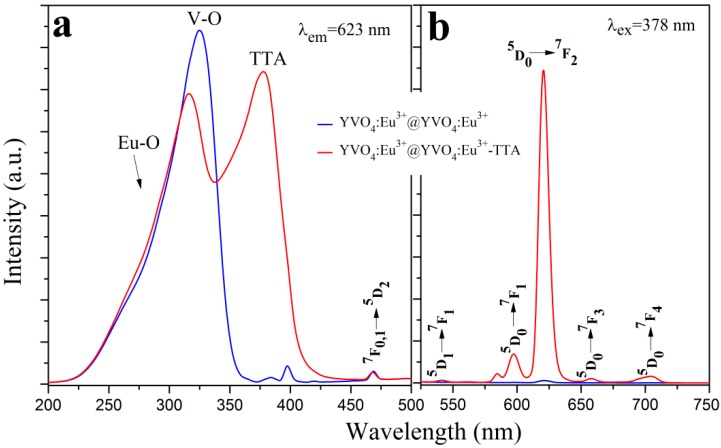
Comparison of the photoluminescence excitation (**a**) and emission (**b**) spectra of the YVO_4_:Eu^3+^@YVO_4_:Eu^3+^ nanoparticles and the YVO_4_:Eu^3+^@YVO_4_:Eu^3+^–TTA inorganic–organic hybrid nanoparticles.

Figure 8 shows the photoluminescence spectra of the YVO_4_:Eu^3+^@YVO_4_:Eu^3+^–TTA inorganic–organic hybrid nanoparticles with different TTA amounts. Figure 9 compares the photoluminescence spectra of the YVO_4_:Eu^3+^@YVO_4_:Eu^3+^ nanoparticles and the YVO_4_:Eu^3+^@YVO_4_:Eu^3+^–TTA inorganic–organic hybrid nanoparticles. Compared with the excitation spectra of the as-synthesized and the annealed YVO_4_:Eu^3+^ nanoparticles (Figure 6a), the YVO_4_:Eu^3+^@YVO_4_:Eu^3+^–TTA inorganic–organic hybrid nanoparticles had a new excitation peak at about 378 nm (Figure 8a), which is a result of the TTA ligands. Consequently, the excitation spectral bandwidth of YVO_4_:Eu^3+^ was notably broadened from the range of 230–330 nm (Figure 6a) to the range of 230–415 nm (Figure 8a). 

At the excitation wavelength of 378 nm, all of the emission peaks (Figure 8b) were from the Eu^3+^ ions, including 542 nm (^5^D_1_→^7^F_1_), 598 nm (^5^D_0_→^7^F_1_), 623 nm (^5^D_0_→^7^F_2_), 655 nm (^5^D_0_→^7^F_3_), and 705 nm (^5^D_0_→^7^F_4_) [31]. This suggests that the YVO_4_:Eu^3+^@YVO_4_:Eu^3+^–TTA inorganic–organic hybrid nanoparticles are efficiently sensitized by TTA ligands. The YVO_4_:Eu^3+^@YVO_4_:Eu^3+^–TTA inorganic–organic hybrid nanoparticles had the strongest photoluminescence when 0.1 mmol of TTA was used for hybridization.

## 4. Conclusions

In summary, YVO_4_ shells were grown on annealed YVO_4_:Eu^3+^ nanoparticle cores, and they were ion exchanged with Eu^3+^ ions, followed by hybridizing with TTA to successfully form the YVO_4_:Eu^3+^@YVO_4_:Eu^3+^–TTA inorganic–organic hybrid nanoparticles. It has been found that annealing process considerably enhances the photoluminescence of the as-synthesized YVO_4_:Eu^3+^ nanoparticles, and the presence of YVO_4_ shells further improves the photoluminescence of the annealed YVO_4_:Eu^3+^ nanoparticles. When the core/shell ratio was 1:0.25, the photoluminescence of the annealed YVO_4_:Eu^3+^ nanoparticles was the strongest. The inorganic–organic hybridization process greatly broadens the photoluminescence excitation spectral bandwidth range of the YVO_4_:Eu^3+^ nanoparticles. After hybridization with TTA, the YVO_4_:Eu^3+^@YVO_4_:Eu^3+^–TTA inorganic–organic hybrid nanoparticles broadened the excitation spectral bandwidth range from 230–330 nm to 230–415 nm, which covers the entire ultraviolet spectrum of the solar light and enables their potential applications in silicon solar cells.
